# Novel Coordination Polymer of Cadmium (II) with L-Tryptophan

**DOI:** 10.3390/ma13102266

**Published:** 2020-05-14

**Authors:** Agnieszka Czylkowska, Małgorzata Szczesio, Anna Pietrzak, Anita Raducka, Bartłomiej Rogalewicz

**Affiliations:** Institute of General and Ecological Chemistry, Faculty of Chemistry, Lodz University of Technology, Zeromskiego 116, 90-924 Lodz, Poland; malgorzata.szczesio@p.lodz.pl (M.S.); anna.pietrzak.1@p.lodz.pl (A.P.); anita.raducka@dokt.p.lodz.pl (A.R.); aeczylko@p.lodz.pl (B.R.)

**Keywords:** coordination polymer, cadmium (II) complex, tryptophan, X-ray structure, Hirshfeld surface, enrichment ratio

## Abstract

A new cadmium (II) polymeric coordination compound with tryptophan (*Trp*) of general formula {[Cd(L-Trp)_2_(H_2_O)Cl]∙(Trp)∙(H_2_O)}_n_ was synthesized. The monocrystals of the investigated complex were obtained using the method of slow evaporation. The crystal and molecular structure was determined. The compound was crystallized in the orthorhombic *P*2_1_2_1_2_1_ space group. The cadmium atom was seven coordinates by two oxygen atoms from one bidentate-chelating carboxylate group of bridging *Trp*, two oxygen atoms from one bidentate-chelating carboxylate group from a monodentate organic ligand, one oxygen atom of water molecule, one nitrogen atom of the amino group from bridging *Trp* and one chlorine atom, which means that every tridentate *Trp* substituent was bridging towards one cadmium atom and bidentate chelating towards one another. The monodentate *Trp* is a zwitterionic molecule. The coordination led to the formation of 1D supramolecular chains entrapping water and *Trp* molecules.

## 1. Introduction

Tryptophan (*Trp*) is an α-amino acid which is very interesting because of its structure. It is a naturally occurring very important amino acid and it is often found at the active sites of proteins. Tryptophan performs many important functions in the human body: allows the synthesis of certain proteins, hormone production and the proper functioning of the nervous system. The human body is not able to synthesize it, so it should be supplied from external sources. Additionally, this organic ligand has many applications e.g., in medicine as a drug, in the development of plant tissues, in nanocomposites or as potentiometric pH sensors [1,2,3,4,5]. For coordination chemistry it is an interesting compound because of the electron donor capability of its amino acid and N-heterocyclic ring. There are many possibilities of coordination to the metal ion. The indole group of tryptophan has great potential for noncovalent interactions in mixed-ligand complexes [6,7]. In some cases, tryptophan links with a metal ion using both an indole and a carboxylic group creating coordination polymers [8,9,10,11,12,13]. These types of compounds are widely studied due to the possibility of their use. They possess flexible chains or nets capable of creating nanoporous channels which may give them potential application. In all cases, the formation of polymeric structures occurs due to the bridging carboxylate group.

Although cadmium has long been regarded as a toxic metal element, cadmium (II) coordination compounds possess a range of novel structures with attractive photochemical and photophysical properties. They have potential applications in photoluminescent diodes, fluorescent probes and optoelectronic devices [14,15].

In this paper the preparation and structural characterization of the coordination polymer of cadmium (II) with tryptophan will be discussed. Interestingly, such coordination led to the formation of a 1D polymeric chain. Moreover, the channels and pockets formed between the neighboring chains included water and *Trp* molecules, respectively. Hirshfeld surface (HS) analysis supported by enrichment ratio calculations were used to identify the intermolecular contacts crucial for the supramolecular assembly of the studied system.

## 2. Materials and Methods

### 2.1. Materials

L−Tryptophan (>98%), CdCl_2_∙2H_2_O (99%) and NaOH (99.9%) were obtained from Merk.

The cadmium (II) complex with tryptophan was prepared by the reaction of L-tryptophan and cadmium chloride (II) dihydrate solution in a stoichiometric ratio. The reaction was carried out under reflux distillation in an aqueous medium. A hot water solution of tryptophan (2.04 g) was mixed with the solution of NaOH (1.68 g). When the tryptophan was completely dissolved, the cadmium chloride (II) (1.83 g) was added. The total mixture volume was 70 mL. The reaction mixture was stirred for six hours. The monocrystals of the investigated complex were obtained using a slow evaporation method. The product was filtered off, washed with a mixture of EtOH and Et_2_O (1:1) *v*/*v* and dried in open air.

### 2.2. Methods

*Single Crystal X-ray analysis.* The crystals were formed of colorless needles. The intensity data was collected on the Rigaku XtaLAB Synergy, Dualflex, Pilatus3 R 300K, (Rigaku Corporation, Tokyo, Japan). Crystal structure refinement was carried out with SHELX [16,17].

CCDC: 1998177 contains the supplementary crystallographic data for this paper. The data is provided free of charge by The Cambridge Crystallographic Data Centre via www.ccdc.cam.ac.uk/structures.

*Hirshfeld surface (HS) and fingerprint plot (FP) analysis.* The CrystalExplorer 17.5 program (University of Western Australia: Crawley, Australia) was used to calculate and generate the Hirshfeld surface of the molecular fragment of the studied system. The molecular geometry applied was derived from the crystal structure. Relevant distances from the HS to the nearest atom (*di*) and the exterior (*de*) to the surface were plotted as scattergrams, namely fingerprints [18,19,20]. Moreover, the enrichment ratios (ER) for meaningful contacts between the atom pairs were calculated [21].

## 3. Results and Discussion

### 3.1. Crystal Structure Determination

**Figure 1:**
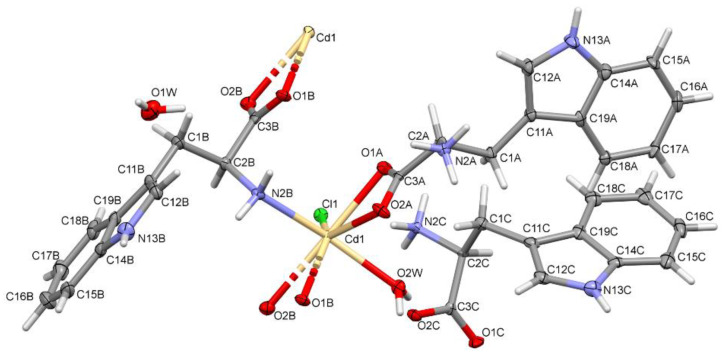
The molecular structure of the complex showing the atom-labeling schemes. Displacement ellipsoids are drawn at the 50% probability level except for the H atoms. Labels A, B and C refer to the symmetrically independent *Trp* molecules.

**Figure 2:**
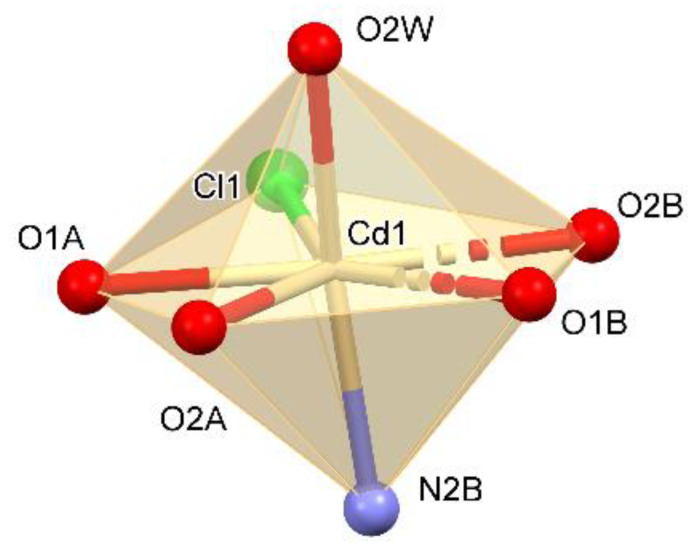
The cadmium coordinate polyhedron.

**Figure 3:**
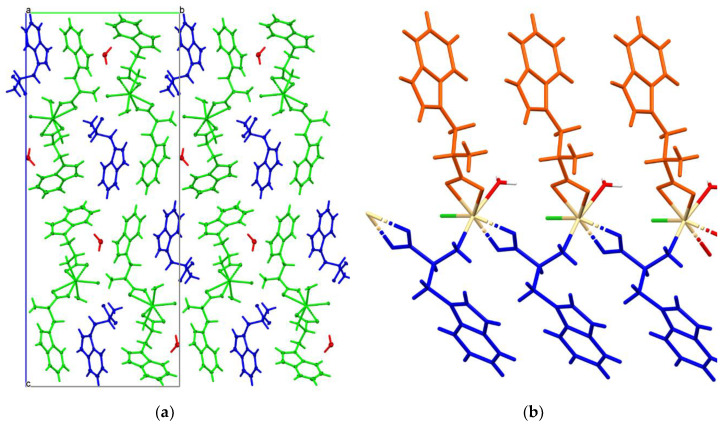
(**a**) Packing of the molecules into the crystal of the compound along the *a* axis; (**b**) the coordination polymer main chain created along the a direction (the zwitterion molecule was marked in orange and the anion in blue).

The structure of the solid-state compound is shown in Figure 1 and its parameters are shown in Table 1. The complex crystallized in the orthorhombic *P*2_1_2_1_2_1_ space group. The asymmetric unit consisted of three tryptophan molecules (molecules A, C—forming zwitterions and B—anion), two water molecules, chlorine anion and a cadmium cation (Figure 1). Two tryptophan molecules and a water molecule existed in the inner coordination sphere of the complex. Additionally, one tryptophan molecule and one water molecule were not bonded with metal ion. The cadmium atom was seven coordinates by two oxygen atoms from one bidentate-chelating carboxylate group of bridging *Trp*, two oxygen atoms from one bidentate-chelating carboxylate group from monodentate organic ligand, one oxygen atom of water molecule, one nitrogen atom of amino group from bridging *Trp* and one chlorine atom, which means that every *Trp* substituent was bridging towards one cadmium atom and bidentate chelating towards one another. The monodentate *Trp* is zwitterionic molecule. Both carboxylate groups of the title compound are bonded unsymmetrically (the Cd–O bond distance is 2.374(4) and 2.528(4) Å (2.349(4) and 2.583(4)) to the central atom. The coordination compound can be described as a pentagonal bipyramid (Figure 2). The anion coordinates formed the 1D polymer through two groups. In contrast, the zwitterion molecule coordinates form the 1D polymer only through the COO group (Figure 3).

**Figure 4:**
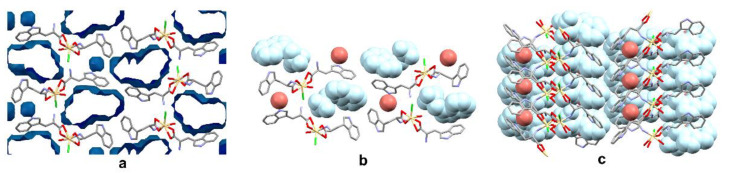
(**a**) The visualization of the channels and the pockets occupied by the tryptophan and water molecules in the studied crystal. Void volumes are based on the contact surface as calculated by Mercury, with a grid step of 0.7 Å and a probe radius of 1.2 Å; the visualization of the assembly of the guest water (red) and the *Trp* (light blue) molecules entrapped between the polymeric chains. View (**b**) along the *a* axis and (**c**) *b* axis. Hydrogen atoms are omitted for clarity.

The polymer scheme was significantly different from the Cambridge Structural Database (CSD, Version 5.41; [22]), because the main chain was created via bridging the *Trp* substituent (with bridging the amino group and the bidentate-chelating carboxylate stabilizing every second polymer mesh). In the title compound each main chain mesh was created from four atoms: one cadmium, two oxygens and one nitrogen. To the best of our knowledge, this is the first known cadmium 1D coordination polymer with such an arrangement of the *Trp* ligand. Moreover, the reported structure contained infinite channels running along the *a* axis and small pockets entrapping the *Trp* and water molecules, respectively (Figure 4). The equatorial plane was defined by the oxygen atoms of the *Trp* ligand (molecules A and B) and chlorine, in the apical position the water molecule and the NH_2_ group of the *Trp* molecule (molecule B) were. Tryptophan (molecule C) molecule and the second water molecule were not involved in coordinating cadmium (Figure 3).

**Figure 5:**
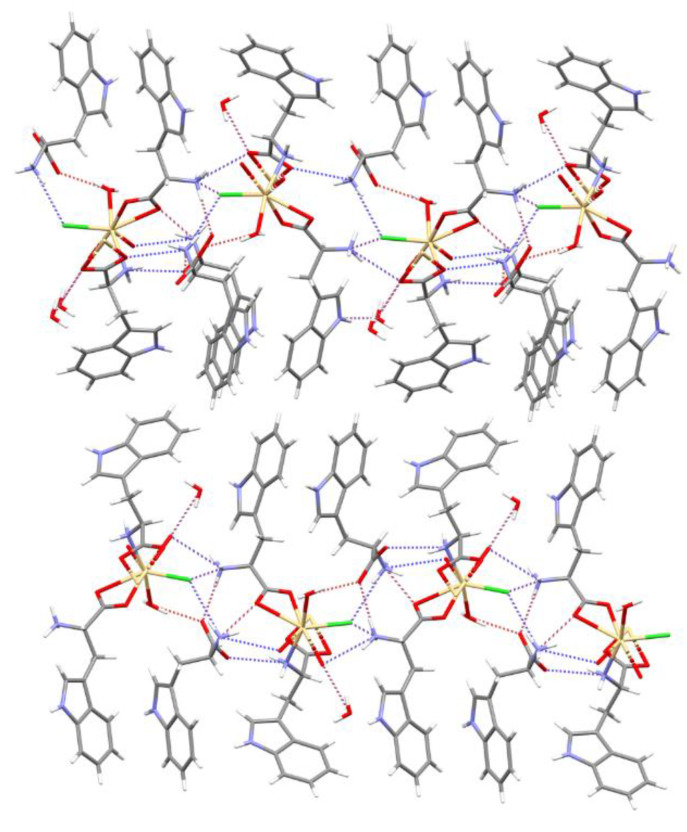
The intermolecular hydrogen bonds (strong bond—red, moderate—blue).

Tryptophan B formed the least hydrogen bonds due to its largest use of atoms in coordinating cadmium (Table 2). The N2A-H⋯O2b hydrogen bond connected the molecules A and B into the infinite C(10) chain according to the definition of Bernstein et al. [23]. In addition, the hydrogen bond N2B-H⋯O1C stabilized the C molecule. All the hydrogen bonds created by the tryptophan molecules stabilized the chains formed parallel to the *b* direction (Figure 5 and Table 2). In the complex existed one water molecule in the inner coordination sphere and one water molecule out of the coordination sphere. The O1W water formed N13a-H⋯O1w-H⋯O2b hydrogen bonds, which formed a chain (C2,2(15)) of tryptophan A and B molecules. In addition, the O2w water, the molecule participating in the coordination, formed the hydrogen bonds of the O2w-H⋯O, respectively. The hydrogen bond O2w-H⋯O1a formed a ring that stabilized the adjacent cations. The second hydrogen binding O2w-H⋯O2c created contact with tryptophan not participating in the cadmium coordination.

**Figure 6:**
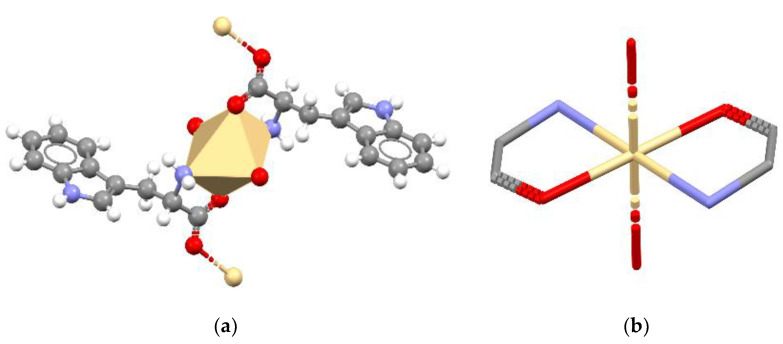
(**a**) The molecular conformation of the complex FUBCIP; (**b**) coordination sphere.

In the CSD database with the FUBCIP refcode, there is a complex with cadmium that contains the Cd (II) ion which is six-coordinates from forming an octahedron. This cadmium integrates with four neutral tryptophan molecules: two *L-Trp* and two *D-Trp* [24]. An additional difference between the cadmium complexes is that in the case of this cadmium ion it was coordinated by N and O atoms (Figure 6a,b). Similarly to the studied complex, FUBCIP forms a polymeric structure. However, FUBCIP forms 2D polymers assembled in layers parallel to the (100) plane. In contrast to the 1D polymeric chain studied by us, the closely packed 2D polymeric planes of FUBCIP do not include any guest molecules in the crystal structure.

### 3.2. Hirshfeld Surface Analysis

The Hirshfeld surface analysis has become a popular supporting tool for the analysis of intermolecular contacts due to its complementarity to typical structure descriptions. Indeed, HS and FP analysis is usually applied for structures formed by hydrogen-bonded molecules or discrete complexes. However, this method may be also applicable to report the polymeric structures. The growing interest of this methodology leads to the continuous development of its applicability [25,26]. The analysis of polymeric structures, by definition, has to be conducted on a selected molecular fragment only. The choice of the fragment for the HS and FP analysis depends on the needs, however we should consider the fact that these would represent not only intermolecular contacts, but also covalent bonds being intersected by the HS. The HS and FP analysis supported by an enrichment ratio (ER) calculation enables the analysis of the propensity of particular species to form interactions within the crystal environment. The enrichment ratio (*ER_XY_*) of a pair of elements (X,Y) allows to compare the actual contacts in the crystal with those computed under the assumption that all contact types have the same probability to form. For pairs of elements having a high propensity to form contacts in the crystal, the enrichment ratio value is expected to be higher than a unity, while pairs which tend to avoid contacts with each other result in *ER_XY_* < 1. Therefore, we decided to apply HS and FP analysis supported by the ER approach to highlight which contacts were statistically favored and were maintaining the crystal packing.

**Figure 7:**
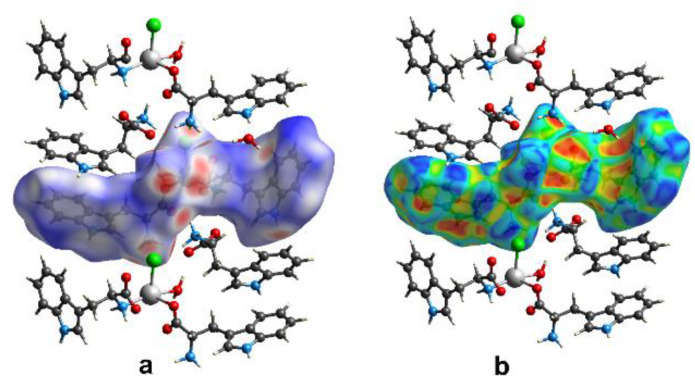
(**a**) Hirshfeld surfaces (HSs) generated for a mer unit of the polymeric chain viewed along the *a* axis. Over the HS *d_norm_*, (**b**) shape index properties are mapped; *d_norm_* is visualized over a fixed color scale of −0.694 (red), 0.430 (white) to 1.332 (blue).

**Figure 8:**
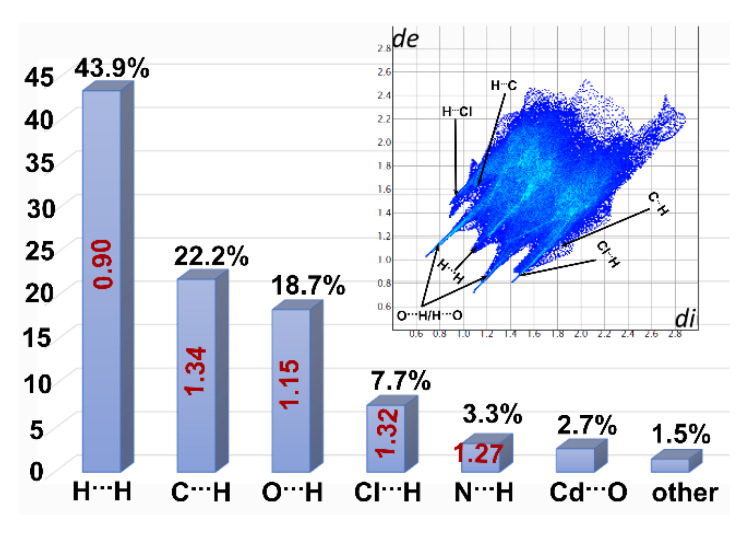
A 2D fingerprint plot (FP) of the title complex with the percentage contributions to the Hirshfeld surface areas for the major intermolecular contacts and their respective enrichment ratio (ER) values-in red; *de* and *di* are the distances to the nearest atom exterior and interior to the surface, respectively; ER values were not computed for the actual contacts’ contribution below 2% and random contacts below 1% as they are not meaningful.

For the studied compound, a 3D Hirshfeld surface (Figure 7a) and the relevant 2D fingerprint map were generated for a mer unit of the polymeric chain. The decomposition of the fingerprint showed that the H⋯H contact comprised 43.9% of the total HS area. However, the respective enrichment ratio value (*ER_HH_* = 0.80) was lower than the unity suggesting that the hydrophobic contacts were indeed disfavored in this structure. Moreover, hydrogen atoms were involved in different types of electrostatic interactions, such as N-H⋯Cl, N-H⋯O, O-H⋯O and C-H⋯π. Additionally, the N-H⋯N hydrogen bonds were represented by a quite low actual contribution (3.3%) of the H⋯N/N⋯H contacts, nevertheless *ER_NH_* = 1.27 indicates that these interactions were also favored. All these X⋯H contacts are clearly visible on the FP scattergram as characteristic spikes (Figure 8) and their overall contribution to the Hirshfeld surface area was 52.1%. The enriched propensity of the supramolecular system of one to form weak C-H⋯π interactions was represented by the significant C⋯H contacts’ contribution of 22.2% with *ER_CH_* = 1.34. These interactions participate in entrapping the guest tryptophan molecule between the polymeric chains. N-H⋯Cl interactions are also favorable in studied systems as indicated by the Cl⋯H contribution (7.7%) accompanied by *ER_ClH_* = 1.32. These interactions between the coordinated Cl atom and the protonated H_3_N^+^ group of the coordinated *Trp* fragment participate in association with the neighboring polymeric chains. It is noteworthy that notably contributing (18.7%) O⋯H contacts have a dual origin. A part of these contacts follows the O-H⋯O interactions between the water molecules and the carbonyl group of the −COO^−^ fragment of the zwitterionic *Trp* molecule. Such interactions associate water and free *Trp* molecules in channels between the polymeric chains. The second type of O⋯H contacts derives from N-H⋯O interactions of the amine and -COO^−^ groups. Moreover, N-H⋯O interaction between the heterocyclic ring of *Trp* and the water molecule was observed. Anyway, the *ER_OH_* value was visibly higher than the unity indicating an increased propensity of the studied system to form diversified types of O⋯H contacts. Cd⋯O contact contribution at the level of 2.7% follows the polymeric chain propagation and reflects the coordination bond between the Cd center and the coordinated oxygen atom. Due to this fact and as the calculated contribution of “random contact” was lower than 1%, this contact was not meaningful for the supramolecular characterization of the studied systems.

Surprisingly, despite the presence of heterocyclic moieties in *Trp* molecules, no meaningful contacts following the π⋯π stacking interactions were observed. Moreover, the shape index mapped on the HS did not reveal a characteristic pattern of the complementary “stamp” and “mold” pairs, features indicating stacking effects, Figure 7b.

## 4. Conclusions

In summary, we reported the synthesis method and the structure of the novel cadmium (II) complex with a tryptophan ligand of general formula {[Cd(L-Trp)_2_(H_2_O)Cl]∙(Trp)∙(H_2_O)}_n_. The unusual coordination mode led to the formation of 1D polymeric chains able to encapsulate not only small molecules like water but also larger species such as the *Trp* molecule. The cadmium atom was seven coordinates. The Hirshfeld surface analysis supported by the enrichment ratio calculation clearly showed that the supramolecular systems were characterized by many types of interatomic contacts. A significant part of the HS area was comprised by H⋯H contacts. Computed *ER_HH_* suggested that their formation was impoverished. In contrast, donor-acceptor interactions i.e., C-H⋯π, N-H⋯Cl, N-H⋯O, O-H⋯O and C-H⋯π were favored for these systems and their overall contribution to the HS was even greater (52.1%) than that of the H⋯H contacts. The analysis excluded any meaningful contribution of dispersive π⋯π stacking interactions in the crystal formation.

## Figures and Tables

**Table 1:** Crystal and structure refinement data for the study complex.

**Crystal Data**	
Chemical formula	C_33_H_39_CdClN_6_O_8_
*M*_r_	795.55
Crystal system, space group	Orthorhombic, *P*2_1_2_1_2_1_
Temperature (K)	100(1)
*a*, *b*, *c* (Å)	5.5754 (2), 15.7300 (2), 38.2694 (6)
*V* (Å^3^)	3356.27 (14)
*Z*	4
Radiation type	Cu *K*
μ (mm^−1^)	6.46
**Data Collection**	
Diffractometer	Rigaku XtaLAB Synergy
Absorption correction	Multi-scan
No. of measured, independent and observed (*I* > 2σ (*I*)) reflections	13477, 5984, 5729
*R*_int_	0.052
(sin θ/λ)_max_ (Å^−1^)	0.636
**Refinement Parameters**	
*R*[*F*^2^ > 2 (*F*^2^)], *wR* (*F*^2^), *S*	0.038, 0.096, 1.04
No. of reflections	5984
No. of parameters	459
No. of restraints	6
H-atom treatment	H atoms treated by a mixture of independent and constrained refinement
Δ〉_max_, Δ〉_min_ (e Å^−3^)	2.53, −0.86

**Table 2:** Hydrogen-bond geometry (Å, °).

*D—H⋯A*	*D—H*	*H⋯A*	*D⋯A*	*D—H⋯A*
N2A—H2AA⋯O2C ^i^	0.89	1.93	2.806 (6)	166
N2A—H2AB⋯Cl1 ^ii^	0.89	2.65	3.121 (5)	115
N2A—H2AB⋯O2B ^ii^	0.89	2.29	2.968 (6)	133
N2A—H2AC⋯Cl1 ^i^	0.89	2.45	3.158 (5)	137
C12A—H12A⋯Cl1 ^ii^	0.93	2.69	3.583 (6)	162
N13A—H13A⋯O1W ^iii^	0.86	2.02	2.864 (8)	167
N2B—H2BB⋯O1C ^i^	0.89	2.19	3.010 (6)	153
N2C—H2CA⋯O2A ^iv^	0.89	2.06	2.786 (6)	139
N2C—H2CA⋯O1B ^v^	0.89	2.40	3.059 (6)	131
N2C—H2CB⋯O1C ^vi^	0.89	1.89	2.766 (6)	167
N2C—H2CB⋯O2C ^vi^	0.89	2.50	3.166 (6)	132
N2C—H2CC⋯Cl1	0.89	2.35	3.227 (5)	168
O1W—HW2⋯O2B	0.84 (1)	2.00 (4)	2.797 (6)	157 (9)
O2W—H2W⋯O1A ^vii^	0.84 (1)	1.95 (2)	2.783 (6)	177 (6)
O2W—H1W⋯O2C	0.84 (1)	1.86 (2)	2.685 (5)	167 (6)

Symmetry codes: ^i^ −*x* + 1, *y* − 1/2, −*z* + 3/2; ^ii^ −*x*, *y* − 1/2, −*z* + 3/2; ^iii^ −*x* − 1, *y* − 1/2, −*z* + 3/2; ^iv^ −*x* + 1, *y* + 1/2, −*z* + 3/2; ^v^ −*x*, *y* + 1/2, −*z* + 3/2; ^vi^ *x* − 1, *y*, *z*; ^vii^ *x* + 1, *y*, *z*.
